# Diffusion-Weighted MRI in Sinonasal Tumors: Diagnostic Insights From a Tertiary Center

**DOI:** 10.7759/cureus.96769

**Published:** 2025-11-13

**Authors:** Kailash Mallineni, Sandya Jayasankaran

**Affiliations:** 1 Radiology, Amrita School of Medicine, Amrita Institute of Medical Sciences and Research Center, Amrita Vishwa Vidyapeetham, Kochi, IND

**Keywords:** apparent diffusion coefficient (adc), diffusion-weighted imaging (dwi), ent - ear nose and throat, head and neck neoplasms, magnetic resonance imaging, onco-imaging, onco-radiology, sinonasal tumor, squamous cell carcinoma (scc), tumor differentiation

## Abstract

This cross-sectional study evaluated the role of magnetic resonance imaging (MRI), including diffusion-weighted imaging (DWI), in differentiating benign from malignant sinonasal lesions. Sinonasal neoplasms are uncommon, comprising approximately three percent of head and neck cancers and one percent of all malignancies. Accurate diagnosis is challenging due to the complex anatomy and overlapping imaging features. Despite advancements in endoscopic imaging and surgery, distinguishing benign from malignant lesions remains difficult. This study aimed to assess whether diffusion-weighted sequences and apparent diffusion coefficient (ADC) values improve the diagnostic accuracy of MRI and to establish an optimal ADC cutoff for malignancy detection.

Seventy-nine patients with histopathologically confirmed sinonasal masses were prospectively evaluated at a tertiary center in India between August 2022 and January 2024. All patients underwent standardized head and neck MRI sequences, including T1-weighted, T2-weighted, short tau inversion recovery (STIR), post-contrast T1, and DWI (b-values 0 and 1000 s/mm²). Mean ADC values were calculated from regions of interest in solid tumor components. Statistical analyses (SPSS version 20, IBM Corp., Armonk, NY) determined diagnostic performance and receiver operating characteristic (ROC) thresholds using histopathology as the reference standard.

An ADC cutoff of 1.37 × 10⁻³ mm²/s provided 97.5% sensitivity and 97.3% specificity, demonstrating near-perfect agreement with histopathology. Among conventional sequences, T2-weighted imaging achieved the highest predictive accuracy, while post-contrast T1 had lower sensitivity. Combined conventional MRI achieved an overall accuracy of 86.8%.

Integrating DWI and ADC mapping into sinonasal MRI protocols significantly enhances diagnostic precision by providing a reliable, noninvasive differentiation between benign and malignant lesions. The established ADC threshold (1.37 × 10⁻³ mm²/s) serves as a robust imaging biomarker that, when used with conventional MRI, supports improved preoperative planning and clinical decision-making for sinonasal tumors.

## Introduction

Sinonasal tumors represent a small but clinically significant subset of head and neck malignancies, accounting for roughly three percent of such cases and about one percent of all cancers [1,2]. Their diagnosis remains challenging due to the complex sinonasal anatomy, subtle early-stage presentation, and overlapping radiological features between benign and malignant lesions [3,4]. Early and accurate differentiation is critical for surgical planning, as treatment strategies differ markedly between inflammatory, benign, and malignant conditions [5].

Over the past three decades, significant advancements in sinonasal disease management have transformed both diagnostic and therapeutic approaches. Enhanced understanding of sinonasal anatomy, combined with the advent of less invasive functional endoscopic sinus surgery (FESS), has greatly expanded access to previously difficult-to-reach regions for biopsy and surgical intervention [3,6].

In India, squamous cell carcinoma and adenocarcinoma are the most common sinonasal malignancies, accounting for approximately fifty percent and thirty percent of cases, respectively [1,7]. The disease shows a male predominance and is most frequently diagnosed between the ages of fifty to seventy years [8]. Established risk factors include environmental pollutants, occupational exposures, tobacco smoke, and human papillomavirus (HPV) infection, which has been implicated in the malignant transformation of sinonasal inverted papillomas [9,10].

Clinical presentation is often nonspecific, with symptoms such as nasal obstruction, facial pain, and epistaxis frequently mimicking benign inflammatory conditions, thereby contributing to delayed diagnosis [5,11]. Advanced disease may present with facial deformity or asymmetry, visual disturbances, and cranial nerve involvement, reflecting extensive local invasion [12].

Cross-sectional imaging plays a central role in evaluating sinonasal lesions. Computed tomography (CT) provides detailed anatomical information on bone destruction and drainage pathways, making it valuable for pre-surgical assessment [1,3]. However, its limited soft-tissue contrast and radiation exposure restrict its utility for detailed tissue characterization [13]. Magnetic resonance imaging (MRI) complements CT by providing superior soft-tissue contrast and multiplanar coverage, making it essential for assessing tumor extension, perineural invasion, and intracranial involvement [1,14]. Despite these advantages, MRI alone often fails to reliably distinguish malignancy from benign inflammatory or infectious processes due to signal overlap [4].

Diffusion-weighted imaging (DWI), an MRI sequence that evaluates the random Brownian motion of water molecules, offers additional microstructural information not visible on conventional MRI. Apparent diffusion coefficient (ADC) values derived from DWI provide quantitative measurements of tissue diffusivity, reflecting tumor cellularity and membrane integrity [15,16]. Malignant tumors typically exhibit restricted diffusion, resulting in lower ADC values, whereas benign lesions display higher ADC averages due to lower cellular density [15]. Previous studies have explored ADC thresholds for differentiating sinonasal lesions; however, published results vary widely, and few have established normative cutoffs applicable to diverse populations [16,17]. Recognizing these diagnostic uncertainties, this study was conducted to evaluate the performance of MRI, including DWI and ADC mapping, in characterizing benign and malignant sinonasal lesions. By correlating MRI findings with histopathology, we aimed to identify specific imaging patterns associated with malignancy and determine an optimal ADC cutoff that enhances diagnostic accuracy. Furthermore, the study sought to assess which MRI sequences most effectively contribute to lesion characterization when used independently and in combination.

This study aims to evaluate the diagnostic utility of magnetic resonance imaging (MRI), including DWI and ADC mapping, for differentiating benign and malignant sinonasal lesions. The broader goal is to improve preoperative diagnostic confidence, guide surgical planning, and reduce unnecessary biopsies by integrating quantitative diffusion metrics into routine MRI protocols [1,17].​​

The objectives are to determine the optimal ADC cutoff value for distinguishing malignant from benign sinonasal lesions, quantitatively analyze ADC values obtained from DWI, and identify which MRI sequences most effectively contribute to lesion characterization. The findings are expected to strengthen diagnostic confidence and support the development of standardized imaging benchmarks for sinonasal pathologies.

## Materials and methods

This prospective observational study was conducted at Amrita Vishwa Vidyapeetham Healthcare Campus, Kochi, India. The study received approval from the Thesis Protocol Review Committee and the Institutional Ethics Committee of the Amrita School of Medicine and was carried out under the supervision of the Department of Radiodiagnosis. All procedures adhered to institutional guidelines.

The study included 79 patients who underwent head and neck MRI with DWI, and in whom the diagnosis of sinonasal lesions was subsequently confirmed by histopathology. Patients without available histopathology results, those with MRI studies performed at outside institutions, those lacking tissue diagnosis, or those ineligible for MRI due to non-compatible implants or claustrophobia were excluded. Histopathological examination was performed mostly preoperatively from endoscopic biopsy specimens. In highly vascular tumors (JNA, AVMs, etc.) and in tumors that were difficult to access endoscopically, biopsy was deferred, and postoperative histopathological examination was performed. Clinical and imaging data were retrieved from electronic medical records and the Picture Archiving and Communication System (PACS).

MRI examinations were performed using a Siemens Biograph mMR 3T scanner equipped with a 32-channel head and neck coil. Standard sequences included axial and coronal T1-weighted, axial, coronal, and sagittal T2-weighted, axial and coronal STIR, and post-contrast 3D T1-weighted images, along with axial DWI using b-values of 0 and 1000 s/mm². Mean ADC values were calculated by manually placing regions of interest (ROIs) in solid tumor areas using Syngo.via software. Mean ADC values were calculated by manually placing ROIs in the solid tumor areas using Syngo.via software. ROIs were placed within the solid portions of the lesion, avoiding necrotic, cystic, hemorrhagic, or calcified regions, as well as adjacent vessels, air, or bone. ROI size ranged from 50 to 100 mm², depending on lesion size. For lesions with a regular shape, a circular ROI was used; for irregularly shaped lesions, a freehand ROI was drawn to best fit the solid portion. Conventional MRI sequences were used to identify solid areas and avoid non-representative regions, strengthening the validity of ADC measurements.

Reproducibility

ROI placement was performed twice by the same radiologist, and intra-observer agreement was assessed using the intraclass correlation coefficient (ICC).

Imaging protocol

Patients were positioned supine during MRI acquisition, with a total scan duration of approximately 35 minutes. Contrast was administered at a dose of 0.2 mL/kg (0.1 mmol/kg) using a mechanical injector at a flow rate of 3.5 mL/second, followed by a 20 mL saline flush. Conventional sequences were analyzed to assess tumor characteristics, including size, extent, internal composition, and enhancement pattern. T2-weighted turbo spin-echo (TSE) and diffusion-weighted (DW) sequences were obtained during free breathing, while T1-weighted STAR-VIBE fat-suppressed sequences were acquired during breath-holding. Axial DWI spin-echo sequences were obtained with a b-value of 1000 s/mm² before contrast injection. ADC maps were generated from DWI images. Circular ROIs measuring 50-100 mm² were manually delineated over solid tumor regions on the ADC maps, with reference to T2-weighted and post-contrast T1-weighted images to avoid cystic or necrotic areas. Quantitative analysis of DW images was performed using Syngo Tissue 4D (Siemens Healthcare, Erlangen, Germany). The mean ADC values were compared across histopathological categories, and receiver operating characteristic (ROC) curve analysis was used to determine the optimal cutoff for differentiating benign and malignant lesions. All MRI images were reviewed by a board-certified radiologist with over fifteen years of experience in head and neck imaging. The radiologist analyzed both conventional and diffusion-weighted sequences using the Syngo multi-modality workstation.

MRI interpretation was performed with the radiologist blinded to the histopathology and final clinical diagnosis. Only anonymized images and basic demographic variables (age, sex) were available at the time of image review.

Histopathology was performed as part of the routine clinical workflow and was not blinded to imaging; however, because histopathology constituted the reference standard, this was unlikely to bias diagnostic accuracy estimates. Thus, the study followed a single-blinded design.

Demographic details, clinical findings, imaging characteristics, surgical data, and histopathology results were recorded using a structured proforma. Data were entered by the lead investigator into a Microsoft 365 Excel master sheet for analysis.

Statistical analysis

Statistical analysis was performed using IBM SPSS Statistics, version 20 (IBM Corp., Armonk, NY). Receiver operating characteristic (ROC) curves were used to determine the optimal ADC cutoff value for differentiating benign and malignant lesions. Diagnostic indices, including sensitivity, specificity, positive predictive value (PPV), negative predictive value (NPV), and overall accuracy, were calculated.

## Results

A total of 79 patients with histopathologically confirmed sinonasal lesions were included in the study. The mean age was 52.4 ± 16.3 years. There was a male predominance, with 54 (68.4%) male patients. The most commonly involved sinuses were the ethmoid sinus (65, 82.3%) and the maxillary sinus (47, 59.5%).

**Figure 1:**
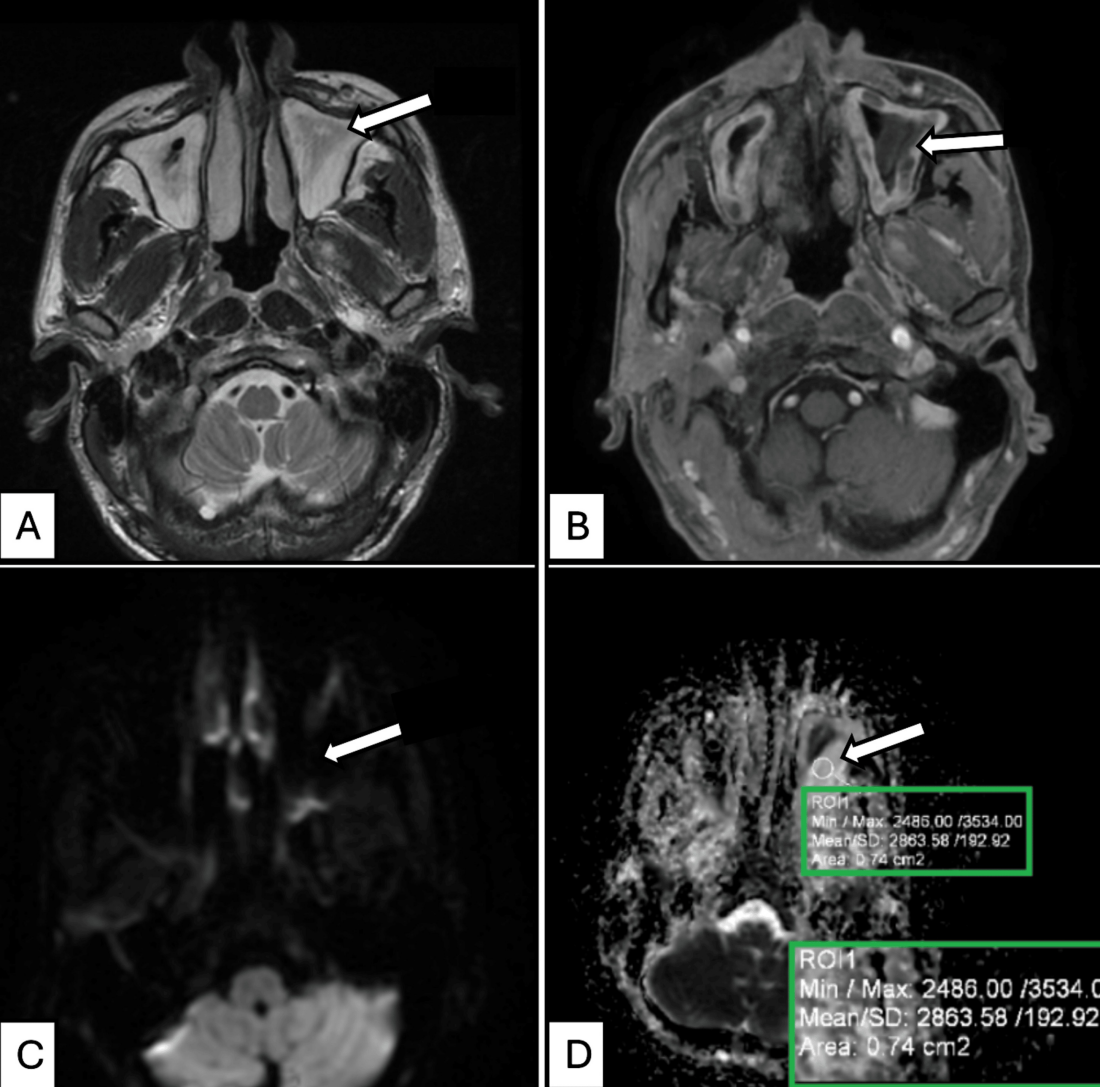
MRI features of chronic sinusitis in a 45-year-old female presenting with headache. (A) Axial T2-weighted image of the sinonasal region shows hyperintense mucosal thickening with isointense fluid within the bilateral maxillary sinuses (arrow marked in the left maxillary sinus). (B) Axial post-contrast T1-weighted image demonstrates enhancing mucosal thickening with non-enhancing fluid in the bilateral maxillary sinuses (arrow marked in the left maxillary sinus). (C) Axial diffusion-weighted image (*b* = 1,000 s/mm²) of the sinonasal region shows hypointense signals within the bilateral maxillary sinus lesions (arrow marked in the left maxillary sinus). (D) Corresponding axial ADC map reveals bright signals with a mean ADC value of 2.863 × 10⁻³ mm²/s (2863.58 × 10⁻⁶ mm²/s) in the left maxillary sinus lesion (arrow marking ROI for ADC measurement). The inset box (bottom right) displays the corresponding quantitative ADC metrics, including minimum, maximum, and mean values, along with the measured ROI area. Histopathology confirmed chronic sinusitis. ADC, apparent diffusion coefficient; ROI, region of interest

Conventional MRI features showed overlapping patterns between benign and malignant lesions. Malignant lesions typically appeared iso- to hypointense on T1-weighted images and T2 isointense and exhibited moderate heterogeneous post-contrast enhancement with necrotic areas and adjacent infiltration. Benign lesions, in contrast, were predominantly hyperintense on T2 and STIR sequences, with well-defined margins and limited invasion of adjacent structures (Figure 1).

**Figure 2:**
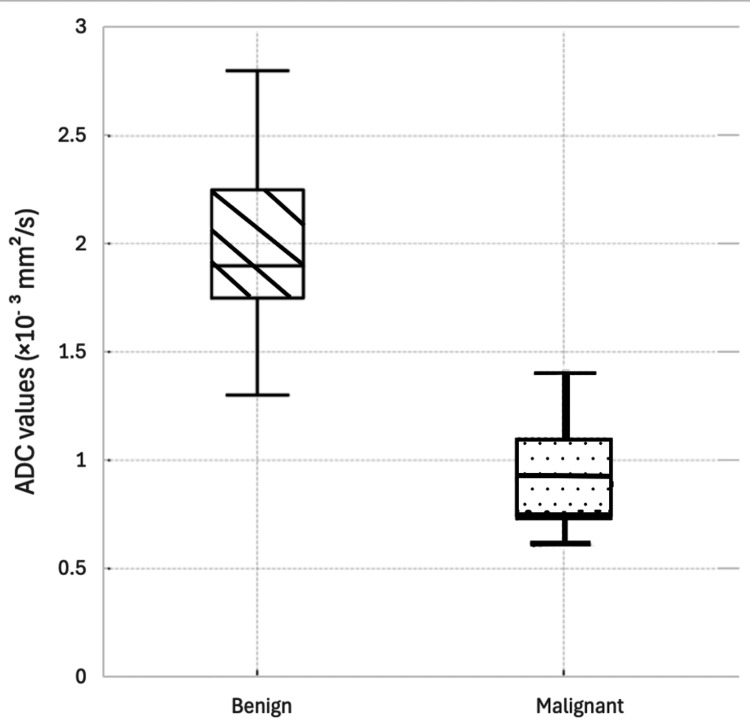
Box-and-whisker chart showing apparent diffusion coefficient (ADC) values of benign and malignant sinonasal tumors. The benign group is represented with lined boxes and the malignant group with dotted boxes.

**Figure 3:**
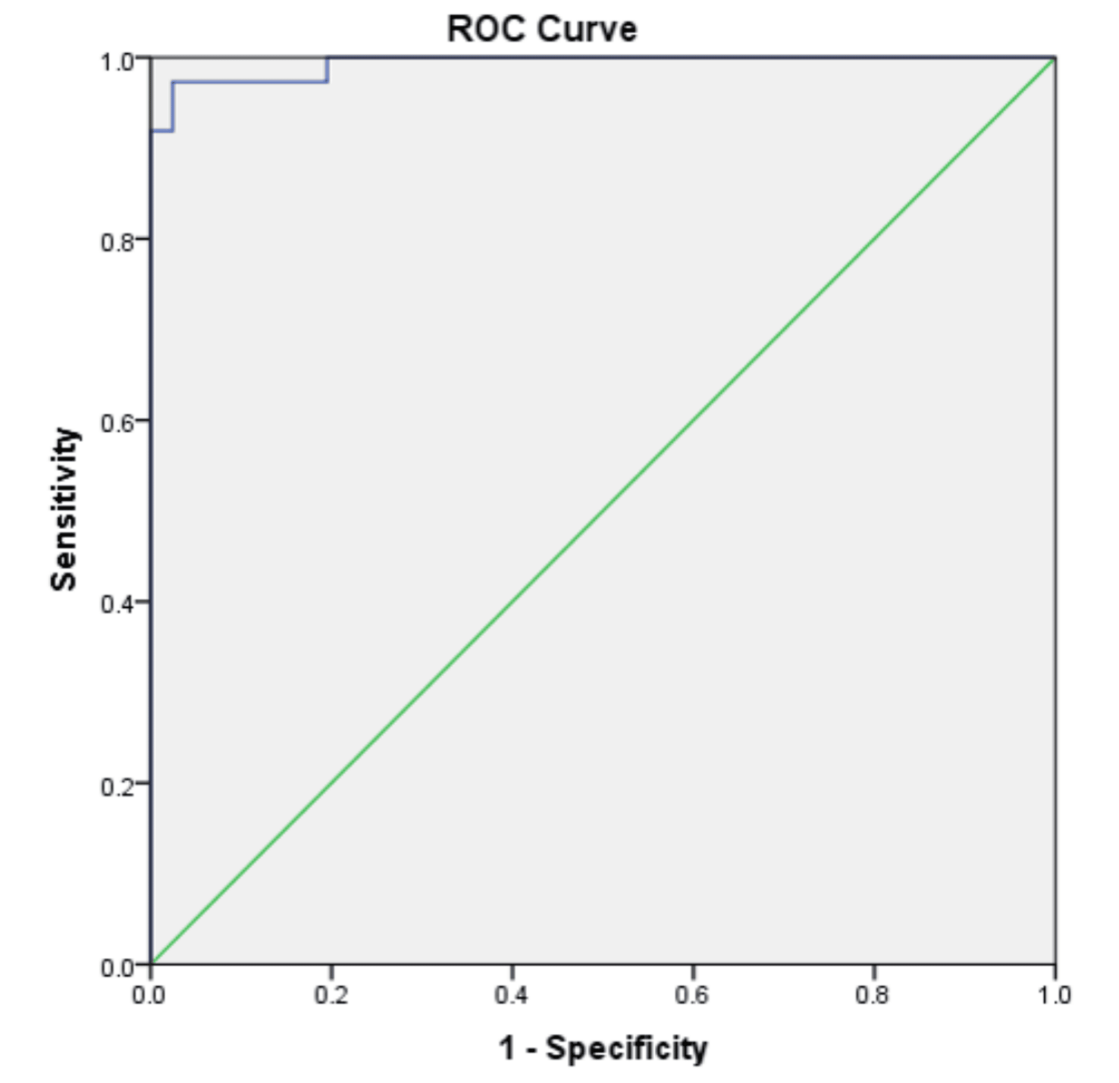
Receiver operating characteristic (ROC) curve for ADC-based differentiation of malignant vs. benign lesions. Using an ADC cutoff of 1.37 × 10⁻³ mm²/s, the curve demonstrates high sensitivity and specificity. ADC, apparent diffusion coefficient

DWI demonstrated superior discriminatory ability between benign and malignant lesions. The mean ADC value was significantly lower in malignant lesions (0.932 ± 0.209 × 10⁻³ mm²/s) compared to benign lesions (2.000 ± 0.325 × 10⁻³ mm²/s) (Figures 2-3). 

**Figure 4:**
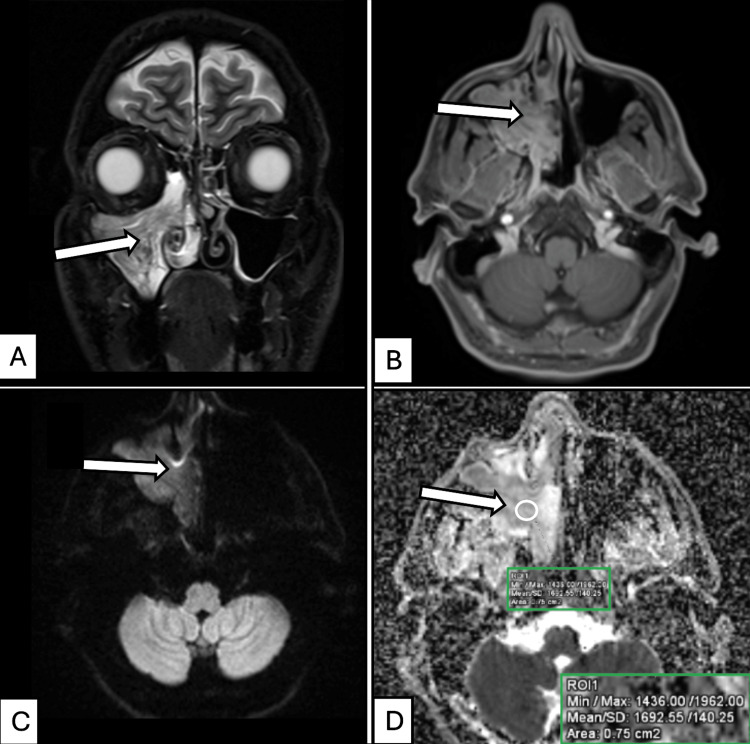
MRI features of Schneiderian papilloma (inverted type) in a 51-year-old male presenting with epistaxis and nasal obstruction. (A) Coronal STIR-weighted image of the sinonasal region shows a STIR-hyperintense lesion with internal isointense bands in the right maxillary sinus and nasal cavity (arrow in right maxillary sinus lesion). (B) Axial post-contrast T1-weighted image demonstrates a moderately heterogeneous enhancing lesion in the same region (arrow in right maxillary sinus lesion). (C) Axial diffusion-weighted image (*b* = 1000 s/mm²) shows an iso- to mildly hyperintense signal within the right maxillary sinus lesion (arrow). (D) Corresponding axial ADC map reveals an isointense signal with a mean ADC value of 1.692 × 10⁻³ mm²/s (1692 × 10⁻⁶ mm²/s) (arrow pointing toward a circular ROI indicating ADC measurement). The inset box (bottom right) displays the corresponding quantitative ADC metrics, including minimum, maximum, and mean values, along with the measured ROI area. Histopathological examination of the endoscopic biopsy confirmed Schneiderian papilloma with focal inverted features. ADC, apparent diffusion coefficient; STIR, short tau inversion recovery

Histopathological examination confirmed 42 benign and 37 malignant lesions. In the present study, the most common malignancy was squamous cell carcinoma (eight cases), the most common benign tumor was sinonasal papilloma (nine cases), and the most frequent benign inflammatory lesion was fungal sinusitis (15 cases) (Figure 4).

**Figure 5:**
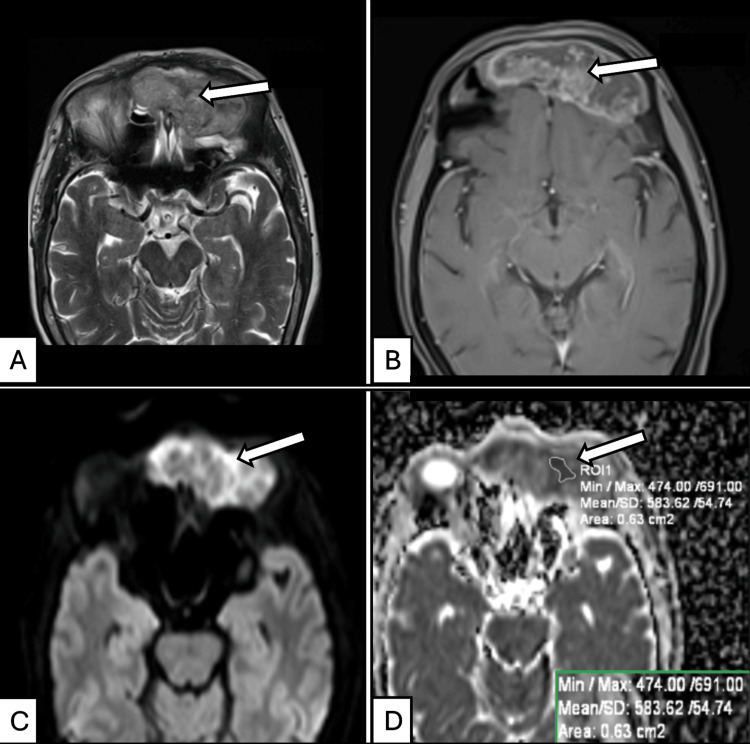
MRI features of moderately differentiated squamous cell carcinoma of the frontal sinuses in a 59-year-old male presenting with epistaxis. (A) Axial T2-weighted image of the sinonasal region shows a lobulated, predominantly isointense lesion involving the bilateral frontal sinuses (arrow in the left frontal sinus lesion). (B) Axial post-contrast T1-weighted image demonstrates moderate, heterogeneous enhancement of the bilateral frontal sinus lesions with central necrosis and no neuroparenchymal involvement (arrow in the left frontal sinus lesion). (C) Axial diffusion-weighted image (*b* = 1000 s/mm²) shows diffusion-bright signals within the bilateral frontal sinus lesions (arrow in the left frontal sinus lesion). (D) Corresponding axial ADC map reveals hypointense signals with diffusion restriction and a mean ADC value of 0.583 × 10⁻³ mm²/s (583 × 10⁻⁶ mm²/s) in the left frontal sinus lesion (arrow marking the ROI for ADC measurement). The inset box (bottom right) displays the corresponding quantitative ADC metrics, including minimum, maximum, and mean values, along with the measured ROI area. Histopathological examination of the endoscopic biopsy confirmed moderately differentiated squamous cell carcinoma. ADC, apparent diffusion coefficient; ROI, region of interest

Signal characteristics on conventional MRI across pathologies are summarized in Table 1 (Figure 5).

**Table 1:** Differentiating T1, T2, STIR, and post-contrast characteristics of various benign and malignant lesions. *Other malignant tumors - Peripheral nerve sheath tumors, phosphaturic mesenchymal tumor, multiphenotypic tumor, basal cell carcinoma, osteosarcoma, clear cell carcinoma, carcinosarcoma, neuroendocrine tumor. T1WI, T1-weighted imaging; T2WI, T2-weighted imaging; STIR WI, short tau inversion recovery weighted imaging

Histopathology	Number of patients	T1WI	T2WI	STIR WI	Pattern of enhancement
Benign inflammatory disease (29 cases, 36.7%)					
Acute and chronic sinusitis	8	Low (7), intermediate (1)	High	High	Mild
Mucocele	3	Low (3)	High	High	Moderate heterogeneous
Fungal sinusitis	15	Low (7), Intermediate (5), High (3)	High (14), Low (1)	Low (1), High (14)	Moderate
Fungal granulomatosis/IgG4	2	Intermediate	High	High	Moderate
Polyposis	1	Intermediate	High	High	Mild
Benign tumors (13 cases, 16.5%)					
Inverted papilloma	9	Intermediate to low SI	Heterogeneous intermediate–high	Heterogeneous intermediate–high	Moderate cribriform
Angiofibroma	2	Intermediate (1), Low (1)	Intermediate to bright	Intermediate to bright	Moderate to intense
AVM	2	Low	Intermediate (with flow voids)	Bright (with flow voids)	Moderate to intense
Malignant tumors (37 cases, 46.8%)					
Squamous cell carcinoma	8	Intermediate (7), Low (1)	Low (1)	Low (2)	Moderate
Adenocarcinoma	1	Low SI	Intermediate (4), High (1)	Intermediate (4), High (1)	Homogeneous
Adenoid cystic carcinoma	6	Intermediate (4), Low (2)	Intermediate	Intermediate (5), High (1)	Homogeneous
Sinonasal undifferentiated carcinoma	4	Intermediate (3), Low (1)	Intermediate	Intermediate (3), High (1)	Moderate
Malignant lymphoma	1	Intermediate	Intermediate	Intermediate	Moderate
Extramedullary plasmacytoma	1	Intermediate	Intermediate	Intermediate	Moderate
Olfactory neuroblastoma	3	Intermediate (1), Low (2)	Intermediate (2), High (1)	Intermediate (2), High (1)	Moderate
Malignant melanoma	3	Intermediate	Intermediate	Intermediate (2), High (1)	Moderate
Rhabdomyosarcoma	1	Intermediate	Intermediate	Intermediate (6)	Moderate
Other malignant tumors*	9	High (2), Low (1)	High (2), Low (1)	High (3)	Moderate
Total	79	-	-	-	-

When composite criteria were applied, conventional MRI achieved a sensitivity of 84.8%, specificity of 89.2%, PPV of 90.7%, NPV of 82.5%, and an overall accuracy of 86.8% in differentiating malignant from benign lesions. Agreement with histopathology was substantial (κ = 0.734, *P* = 0.001).

When ADC criteria were applied, ROC analysis confirmed high diagnostic accuracy. An ADC threshold of 1.37 × 10⁻³ mm²/s achieved a sensitivity of 97.5%, specificity of 97.3%, and an area under the curve (AUC) of 0.993, indicating near-perfect agreement with histopathology (κ = 0.949, *P* = 0.001). At this cutoff, the diagnostic indices were as follows: sensitivity 97.6%, specificity 97.3%, PPV 97.6%, NPV 97.3%, and overall accuracy 97.4%.

These findings demonstrate that ADC measurement not only provides quantitative differentiation between benign and malignant sinonasal lesions but also enhances diagnostic confidence, particularly when conventional MRI features are equivocal.

Summary of diagnostic performance

Conventional MRI alone: Accuracy 86.8%, κ = 0.734

DWI (ADC cutoff 1.37 × 10⁻³ mm²/s): Accuracy 97.4%, AUC 0.993, κ = 0.949

Overall, DWI with quantitative ADC evaluation substantially outperformed conventional MRI in differentiating sinonasal lesions, establishing itself as a reliable, noninvasive diagnostic tool in routine clinical practice.

Mean ADC values by histology are summarized in Table 2.

**Table 2:** Mean ADC values of various benign and malignant lesions. ADC, apparent diffusion coefficient

Malignant type	No.	Mean ± SD	Minimum to maximum	Benign type	No.	Mean ± SD	Minimum to maximum
Squamous cell carcinoma	8	1.046 ± 0.165	0.82-1.2	Sinonasal papillomas	9	1.86 ± 0.26	1.5-2.4
Adenocarcinoma	1	0.985	0.985	JNA	2	2.5 ± 0.62	2.1-2.9
Adenoid cystic carcinoma	6	1.082 ± 0.35	0.64-1.71	AVM	2	2.06 ± 0.36	1.8-2.3
Sinonasal undifferentiated carcinoma	4	0.9 ± 0.3	0.65-1.3	Acute sinusitis	5	1.9 ± 0.2	1.6-2.2
Malignant lymphoma	1	0.67	0.67	Chronic sinusitis	3	1.9± 0.12	1.7-2.17
Extramedullary plasmacytoma	1	0.59	0.59	Mucocele	3	1.94 ± 0.19	1.8-2.1
Olfactory neuroblastoma	3	1.07 ± 0.28	0.76-1.322	Fungal sinusitis	15	2.1± 0.23	1.7-2.6
Malignant melanoma	3	0.7 ± 0.09	0.6-0.8	Polyposis	1	2.29	2.29
Rhabdomyosarcoma	1	0.564	0.564	IgG4	1	1.39	1.39
Peripheral nerve sheath tumor	1	0.996	0.996	Fungal granulomatous disease	1	1.91	1.91
Phosphaturic mesenchymal tumor	1	0.939	0.939				
Multiphenotypic carcinoma	2	0.946 ± 0.13	0.85-1.03				
Basal cell carcinoma	1	1.05	1.05				
Osteosarcoma	1	1.01	1.01				
Clear cell carcinoma	1	1.04	1.04				
Carcinosarcoma	1	0.88	0.88				
Neuroendocrine carcinoma	1	0.88	0.88				

## Discussion

This study demonstrates that DWI with ADC mapping significantly enhances the differentiation between benign and malignant sinonasal lesions compared to conventional MRI sequences. MRI findings of inflamed sinonasal mucosa showed low signal intensity (SI) on T1-weighted imaging and high SI on T2-weighted imaging, reflecting high water content, consistent with El-Gerby and El-Anwar [17]. Notably, cases of fungal sinusitis and mucocele demonstrated low SI on T2-weighted imaging, likely due to the presence of minerals such as calcium, iron, and magnesium within fungal hyphae, producing hypointense or signal-void regions. These distinct MRI characteristics highlight the diagnostic value of recognizing fungal patterns [17].

Incorporating quantitative ADC measurements significantly improved diagnostic accuracy compared to conventional MRI alone. The ADC cutoff of 1.37 × 10⁻³ mm²/s achieved 97.5% sensitivity, 97.3% specificity, and 97.4% accuracy, consistent with previous reports. El-Gerby and El-Anwar reported a cutoff of 1.2 × 10⁻³ mm²/s (90% accuracy, 100% sensitivity, and 88.4% specificity), Sasaki et al. 0.84 × 10⁻³ mm²/s (75% sensitivity, 94% specificity), and Razek et al. 1.53 × 10⁻³ mm²/s (93% accuracy, 94% sensitivity, and 92% specificity) [15-17]. These findings confirm the clinical relevance of ADC as a noninvasive biomarker aiding preoperative assessment and reducing unnecessary biopsies.

Our research also delved into the ADC values of specific malignant types, with findings like lower ADC values in rhabdomyosarcoma (0.564 × 10⁻³ mm²/s) and lymphoma (0.67 × 10⁻³ mm²/s), aligning with literature that associates reduced ADC with increased tumor cellularity. Yet, the limited case numbers for these malignancies in our study call for cautious interpretation of these results [15-17].

Conventional MRI sequences, particularly T2-weighted imaging, provided useful contrast but limited specificity due to overlapping signal characteristics among inflammatory, benign, and malignant lesions [4]. The addition of ADC values offered an objective quantitative parameter that improved lesion classification, especially in diagnostically challenging cases.

This study contributes valuable data from the Indian subcontinent, where literature on MRI-based sinonasal tumor differentiation remains limited. The manual ROI delineation and strong correlation with histopathology reinforce the validity of the findings. Integration of DWI with routine MRI protocols is recommended for comprehensive sinonasal lesion evaluation.

Limitations

Despite these strengths, several limitations warrant consideration. The sample size, though adequate for the primary analysis, was limited for rare histopathologic subtypes, restricting subtype-specific conclusions. Although we provided standardized guidelines for manual ROI placement, interobserver reproducibility of ROI measurements was not assessed in this study, which is a limitation and should be addressed in future research. Finally, as a single-center study, the results require validation across larger, multi-institutional cohorts.

## Conclusions

This study confirms that DWI with ADC mapping enhances the differentiation between benign and malignant sinonasal lesions when compared to conventional MRI alone. Incorporating quantitative diffusion metrics into routine sinonasal MRI protocols offers a reliable, noninvasive tool to support clinical decision-making and surgical planning.

Despite these promising findings, certain limitations exist, including the limited sample size for less common histopathologic subtypes and potential observer variability in manual region-of-interest delineation. Future multicenter studies with automated ADC measurement techniques are recommended to further validate these findings and refine diagnostic protocols for sinonasal lesions.
